# TWEAK Promotes Peritoneal Inflammation

**DOI:** 10.1371/journal.pone.0090399

**Published:** 2014-03-05

**Authors:** Ana Belen Sanz, Luiz Stark Aroeira, Teresa Bellon, Gloria del Peso, Jose Jimenez-Heffernan, Beatriz Santamaria, Maria Dolores Sanchez-Niño, Luis Miguel Blanco-Colio, Manuel Lopez-Cabrera, Marta Ruiz-Ortega, Jesus Egido, Rafael Selgas, Alberto Ortiz

**Affiliations:** 1 Laboratory of Nephrology, IIS-Fundacion Jimenez Diaz, Madrid, Spain; 2 Department of Immunology, Instituto de Investigación Biomédica de Vigo (IBIV), Vigo, Pontevedra, Spain; 3 Department of Nephrology, IDIPAZ, Madrid, Spain; 4 REDinREN, Madrid, Spain; 5 Hospital Universitario de la Princesa, Madrid, Spain; 6 Centro Biologia Molecular Severo Ochoa, Madrid, Spain; 7 Universidad Autonoma de Madrid, Madrid, Spain; 8 IRSIN, Madrid, Spain; French National Centre for Scientific Research, France

## Abstract

Peritoneal dialysis (PD) is complicated by peritonitis episodes that cause loss of mesothelium and eventually sclerosing peritonitis. An improved understanding of the molecular contributors to peritoneal injury and defense may increase the therapeutic armamentarium to optimize peritoneal defenses while minimizing peritoneal injury. There is no information on the expression and function of the cytokine TWEAK and its receptor Fn14 during peritoneal injury. Fn14 expression and soluble TWEAK levels were measured in human PD peritoneal effluent cells or fluids with or without peritonitis. Fn14 expression was also analyzed in peritoneal biopsies from PD patients. Actions of intraperitoneal TWEAK were studied in mice in vivo. sTWEAK levels were increased in peritoneal effluent in PD peritonitis. Effluent sTWEAK levels correlated with the number of peritoneal macrophages (r = 0.491, p = 0.002). Potential TWEAK targets that express the receptor Fn14 include mesothelial cells and macrophages, as demonstrated by flow cytometry of peritoneal effluents and by analysis of peritoneal biopsies. Peritoneal biopsy Fn14 correlated with mesothelial injury, fibrosis and inflammation, suggesting a potential deleterious effect of TWEAK/Fn14. In this regard, intraperitoneal TWEAK administration to mice promoted peritoneal inflammation characterized by increased peritoneal effluent MCP-1, Fn14 and Gr1^+^ macrophages, increased mesothelial Fn14, MCP-1 and CCL21 expression and submesothelial tissue macrophage recruitment. Taken together these data suggest that the TWEAK/Fn14 system may promote inflammation and tissue injury during peritonitis and PD.

## Introduction

Peritonitis is a potentially devastating disease occurring in the context of abdominal visceral injury, cirrhosis, peritoneal dialysis (PD) and others. PD is a renal replacement therapy modality that is marred by episodes of bacterial infection, leading to localized inflammation evidenced as peritonitis [1]. Moreover, dialysis solutions can themselves induce sterile peritoneal inflammation [2], [3]. PD represents an interesting human model of inflammation since the technique allows repeated non-invasive access to the peritoneal cavity, allowing both monitoring of the inflammatory process as well as therapy by local delivery of drugs [4]. Peritoneal inflammation is characterized by local upregulation of several cytokines, macrophage recruitment, and collagen synthesis by mesothelial cells and fibroblasts leading to loss of peritoneal membrane integrity and fibrosis. Both acute and chronic peritoneal inflammation may lead to PD technique failure [5]. In some cases PD-associated chronic peritoneal inflammation may result in sclerosing peritonitis [6]. Sclerosing peritonitis is a fatal form of peritoneal inflammation characterized by a fibrous thickening of the peritoneum. Understanding the role of the different players involved may help design strategies to limit inflammation-mediated tissue injury without compromising antibacterial defenses.

Tumor necrosis factor-like weak inducer of apoptosis (TWEAK, TNFSF12) is a member of the TNF superfamily of structurally-related cytokines. TWEAK may modulate cell death, proliferation, inflammation and angiogenesis [7]–[10]. Fibroblast growth factor-inducible 14 (Fn14) is the functional TWEAK receptor. Fn14 expression is strongly induced during tissue injury, repair and remodeling [11]. Cells can express full-length membrane-anchored TWEAK (mTWEAK) and secrete a soluble form (sTWEAK), and both bind and activate Fn14 [11]. sTWEAK levels in serum/plasma or urine may have biomarker value in inflammatory diseases, such as atherosclerosis, lupus nephritis and chronic kidney disease [12]–[14]. The role of TWEAK in inflammation has been described in the central nervous system, cardiovascular injury and kidney disease [15]–[20], but there is no information on the expression and role of TWEAK and Fn14 during human infection or peritoneal inflammation.

We have now explored the expression of TWEAK and Fn14 during human peritoneal infection and in vivo effects of TWEAK on peritoneal inflammation. We report that sTWEAK levels are increased in effluents from PD patients with peritonitis and correlate with effluent macrophage number. Fn14 expression is also increased in cells from peritonitis effluents and local tissue Fn14 expression correlates with mesothelial injury in human peritoneal biopsies. Finally, intraperitoneal TWEAK injection in mice promotes peritoneal inflammation and increases macrophages in peritoneal effluents and in the peritoneal tissue. These results support a role of TWEAK in peritoneal injury and inflammatory cell recruitment and suggest that TWEAK may be a biomarker of peritoneal inflammation during PD.

## Materials and Methods

### Human samples

Human samples were obtained following a protocol approved by the IDIPAZ Ethics Committee and informed consent was obtained. Peritoneal effluent samples were obtained from patients undergoing chronic PD in the course of a peritonitis episode (n = 14) or from stable PD patients without peritonitis in the prior 3 months and without any other cause systemic inflammation (Non-peritonitis group; n = 8). The characteristics of study participants are presented in **Tables 1** **and** **2**. Human peritoneum biopsy samples were obtained at the time of transplantation or abdominal surgery from 9 patients without evidence of systemic inflammation (**Table 3**). PD patients were stable at the time of biopsy, with no recent peritonitis. An experienced pathologist (JJH) scored the biopsies blinded as to the nature of the sample and following a previously established peritoneal injury score. This score assesses 3 items (mesothelial integrity, peritoneal fibrosis and peritoneal inflammation) in a 0–3 scale for a total possible maximum score of 9 for most severely injured samples [21]. In this regard, in the present manuscript, the published score was modified in order to assign a score of 3 (rather than 0) to the most severe mesothelial injury (loss of mesothelium) and a score of 0 (rather than 3) to mesothelial integrity.

**Table 1:** Clinical characteristics of PD patients with peritonitis providing peritoneal effluent samples.

Patient	Age (years)	Sex	Cause of ESRD	Peritonitis number for the patient	Culture results	Duration of peritonitis (days)	Catheter removal	PD vintage at time of peritonitis (months)
1	76	Male	Nephroangiosclerosis	7^th^	*Enterococcus faecalis*	2	Yes	62
				8^th^	*Klebsiella*	2	No	65
				9^th^	*Klebsiella*	2	No	66
2	59	Male	Glomerulonephritis	3^rd^	*Escherichia coli*	7	No	30
3	64	Male	Nephroangiosclerosis	1^st^	*Streptococcus salivarius*	3	No	11
4	72	Female	Interstitial	1^st^	*Streptococcus*	2	No	19
5	30	Female	Vasculitis	1^a^	*Campylobacter fetus*	2	No	6
6	71	Male	Nephroangiosclerosis	3^rd^	*Escherichia coli*	16	Yes	13
7	84	Male	Unknown	1^st^	*Bacteroides fragilis*	4	No	5
8	71	Male	Glomerulonephritis	2^nd^	*Streptococcus salivarius*	2	No	13
				3^rd^	*Staphylococcus coagulase negative*	5	No	15
9	74	Male	Unknown	1^st^	*Staphylococcus epidermidis*	2	No	9
10	48	Male	Unknown	1^st^	Sterile	3	No	-
11	48	Male	Unknown	1^st^	*Streptococcus*	4	No	0.5
12	50	Female	Unknown	4^th^	*Klebsiella*	5	No	37
13	25	Male	Interstitial	1^st^	*Staphylococcus aureus*	4	No	3
14	65	Female	Unknown	2^nd^	*Candida*	7	Yes	12

ESRD: End-stage renal disease.

**Table 2:** Clinical characteristics of stable PD patients without peritonitis in the prior 3 months providing peritoneal effluent samples.

Patient	Age	Sex	Cause of ESRD
1	30	Female	Vasculitis
2	30	Female	Unknown
3	60	Female	Polycystic kidneys
4	57	Male	Nephroangiosclerosis
5	76	Male	Diabetes type II
6	58	Female	Interstitial
7	29	Male	Glomerulonephritis
8	29	Male	Glomerulonephritis

ESRD: End-stage renal disease.

**Table 3:** Clinical characteristics of patients providing peritoneal biopsy samples.

Patient	Age (years)	Sex	Cause of ESRD	PD vintage (months)	MTC Cr (ml/min)	4.25% glucose UF (ml/4 h)	Mesothelial integrity	Fibrosis	Chronic inflammation	Peritoneal injury score
1	53	M	ND	Non-ESRD	ND	ND	0	1	0	1
2	59	M	Tubulointerstitial Nephritis	HD	ND	ND	0	0	0	0
3	68	M	Glomerulonephritis	12	8.7	600	0	0	0	0
4	27	M	Unknown	8	10.4	900	0	1	0	1
5	55	F	Glomerulonephritis	21	8.9	600	2	0	0	2
6	34	M	Glomerulonephritis	4	6.3	870	2	1	2	5
7	25	F	Glomerulonephritis	52	6.6	850	3	2	2	7
8	32	M	Interstitial	23	13.7	480	2	1	1	4
9	50	F	Unknown	37	10.8	310	2	2	2	6

ND: no data. ESRD: End-stage renal disease.

HD: hemodialysis. M: male, F: female.

### ELISA

sTWEAK levels in human peritoneal effluent were determined with a commercially available enzyme-linked immunosorbent assay (ELISA) kit (BMS2006INST; Bender MedSystems, Vienna, Austria). MCP-1 levels in murine peritoneal lavages were measured by commercial ELISA (BD Pharmingen, NJ).

### Mesothelial cell cultures

The study was approved by the clinical ethics committee of IIS-Fundación Jiménez Díaz and written informed consent was obtained. Human omental mesothelial cells (HOMC) were obtained from omentum from 6 non-PD patients who underwent unrelated elective abdominal surgery [22], [23].

### Western blot

Cell samples were homogenized in lysis buffer (50 mM TrisHCl, 150 mM NaCl, 2 mM EDTA, 2 mM EGTA, 0.2% Triton X-100, 0.3% NP-40, 0.1 mM PMSF and 1 µg/ml pepstatin A) then separated by 12% SDS-PAGE under reducing conditions [20]. After electrophoresis, samples were transferred to PVDF membranes (Millipore), blocked with 5% skimmed milk in PBS/0.5% v/v Tween 20 for 1 hr, washed with PBS/Tween, and incubated with rabbit polyclonal anti-Fn14 (1∶1000, Cell Signaling, Danvers, MA) and rabbit polyclonal anti-TWEAK (1∶500, Santa Cruz Biotechnology, Santa Cruz, CA) diluted in 5% BSA. Blots were washed with PBS/Tween and incubated with appropriate horseradish peroxidase-conjugated secondary antibody (1∶2000, Amersham, Aylesbury, UK). After washing with PBS/Tween blots were developed with the chemiluminescence method (ECL) (Amersham). Blots were then probed with mouse monoclonal anti-α-tubulin antibody (1∶2000, Sigma) and levels of expression were corrected for minor differences in loading.

### Fn14 immunofluorescence colocalization in human peritoneum biopsies

The general procedures have been previously described [24]. Antigenic epitope retrieval was performed in 3 µm thick sections of paraffin-embedded tissue using a PTlink device (with a high pH solution, 95°C, 20 min). Tissue slides were incubated overnight at 4°C with the primary antibodies, rabbit polyclonal anti-Fn14 (1∶100, Cell Signaling) and mouse monoclonal anti-cytokeratin 8 (1∶60, Santa Cruz Biotechnology) or mouse monoclonal anti-CD68 (Dako Diagnostics, Barcelona, Spain). Slides were then washed with PBS and incubated with anti-rabbit Alexa488 and anti-mouse Alexa 633 conjugated secondary antibodies (1∶300, Invitrogen, Carlsbad, CA).

### Fn14 immunohistochemistry

In human peritoneum biopsies antigenic epitope retrieval was performed in 3 µm thick sections of paraffin-embedded tissue using a PTlink device (with a high pH solution, 95°C, 20 min). Endogenous peroxidase was blocked by incubation in 3% H2O2/methanol (1∶1) at 25°C for 30 minutes [25]. Slides were incubated in PBS with 4% bovine serum albumin (BSA) and 1% horse serum, for 1 hour at 37°C to reduce nonspecific background staining, and then incubated overnight at 4°C with rabbit polyclonal anti-Fn14 (1∶100, Cell Signaling) in PBS, 4% BSA, 1% serum. After washing with PBS, sections were incubated with a secondary biotin-labeled antibody, washed and developed with AB streptavidin-complex. Finally, sections were washed and stained with DAB (Dako Diagnostics). Sections were counterstained with Carazzi's hematoxylin. Negative controls included incubation with isotype IgG. Fn14 staining was evaluated by a quantitative scoring system, Image-Pro Plus software (Media cybernetics, MD) in 12 randomly selected fields (x20) per sample by an observer blinded as to eh nature of the samples.

In murine samples antigenic epitope retrieval was performed in 5 µm thick sections of paraffin-embedded tissue using a PTlink device (with a high pH solution, 95°C, 20 min). Tissue slices were incubated for 30 min at room temperature with the primary antibody, rabbit polyclonal anti-Fn14 (1∶100, Cell Signaling), goat polyclonal anti-MCP-1 (1∶500, Santa Cruz), goat polyclonal anti-CCL21 (1∶150, R&D Systems, Minneapolis, MN) and rat polyclonal anti-F4/80 (1∶10000, Serotec) [20]. For immunohistochemical staining Envision FLEX + visualization system was used, in a DAKO Autostainerplus platform. Tissue sections were subsequently counterstained with hematoxylin. Same sections were incubated without the primary antibody as negative controls.

### Animal model

Studies were conducted in accord with the NIH Guide for the Care and Use of Laboratory Animals. C57BL6 female mice (12- to 14-week-old) (IFFA-CREDO, Barcelona, Spain) received 0.75 µg/human recombinant TWEAK (Millipore, Billerica, MA) or saline (n = 5 per group) intraperitoneally. Mice were killed 4 h or 24 h after injection. The dose of TWEAK was calculated based on in vitro experiments for an extracellular volume of 7.5 ml/mouse [19].The dose had been validated in prior animal experiments, where the time course of systemic TWEAK actions were explored with focus on the kidney. To determine peritoneal production of chemokines, the peritoneal cavity was lavaged with two ml saline immediately after mice were euthanised. The solution (murine peritoneal lavage) was centrifuged and the supernatant was separated into small aliquots and stored at −80°C and the pellet containing cells was used for flow cytometry analysis and for RT-PCR. After sacrifice parietal peritoneum was collected, fixed in neutral-buffered formalin and embedded in paraffin [26].

### Flow cytometry

Human peripheral blood mononuclear cells, human peritoneal effluent cells and murine peritoneal effluent cells were analyzed by flow cytometry.

Cells were isolated from PD effluents by centrifugation at 500 g for 15′ at 4°C. PBMCs from healthy control donors were prepared by Ficoll-Hypaque (Amersham Pharmacia Biotech, Sweden) density gradient centrifugation. The following mAb were used for flow cytometry analysis of surface molecules: CD3-PerCP, CD14-FITC, CD56-APC (BD Biosciences) and Fn14-PE (eBioscience). Cells were pre-incubated with 50 µg/mL human IgG to prevent binding to FcR and stained according to standard protocols. Analysis was performed in a FACScalibur cytometer with ProQuest software (BD Bioscience).

Cells from murine peritoneal effluent were analyzed by flow cytometry using the following antibodies: anti-mouse CD4 PE and Alexa Fluor® 488-labelled anti-mouse CD8a for T cells. F4/80 PE-Cy7, Gr1 APC, CD14 FITC and CD11b PE for macrophages (BD Biosciences, San Diego, USA). Macrophages were defined as CD11b+F4/80+ cells and Gr1+ macrophages as CD11b+F4/80+Gr1+ cells. Neutrophils were defined as Gr1+/F4/80- cells.

### RNA extraction and Real-Time Polymerase Chain Reaction

Total RNA was extracted from cells and tissue by the TRI Reagent method (Sigma) and 1 µg of RNA was reverse transcribed with High Capacity cDNA Archive Kit (Applied Biosystems, Foster City, CA). Pre-developed primer and probe assays for Fn14, and GAPDH (human) were from Applied (Applied Biosystems). Quantitative PCR was performed by 7500 Real Time PCR System with the Prism 7000 System SDS Software (Applied Biosystems) and RNA expression of different genes was corrected for GAPDH [27].

### Statistical analysis

Non-normally distributed variables were expressed as median (interquartile range), and normally distributed variables were expressed as mean ± SEM. p<0.05 was considered to be statistically significant. Mann–Whitney tests were used to compare the results between two groups. Spearman rank correlation was used to determine correlations between two variables. Statistical analysis was performed with SPSS software package version 11.0.

## Results

### sTWEAK concentrations are increased in peritoneal effluent from PD peritonitis

**Figure 1:**
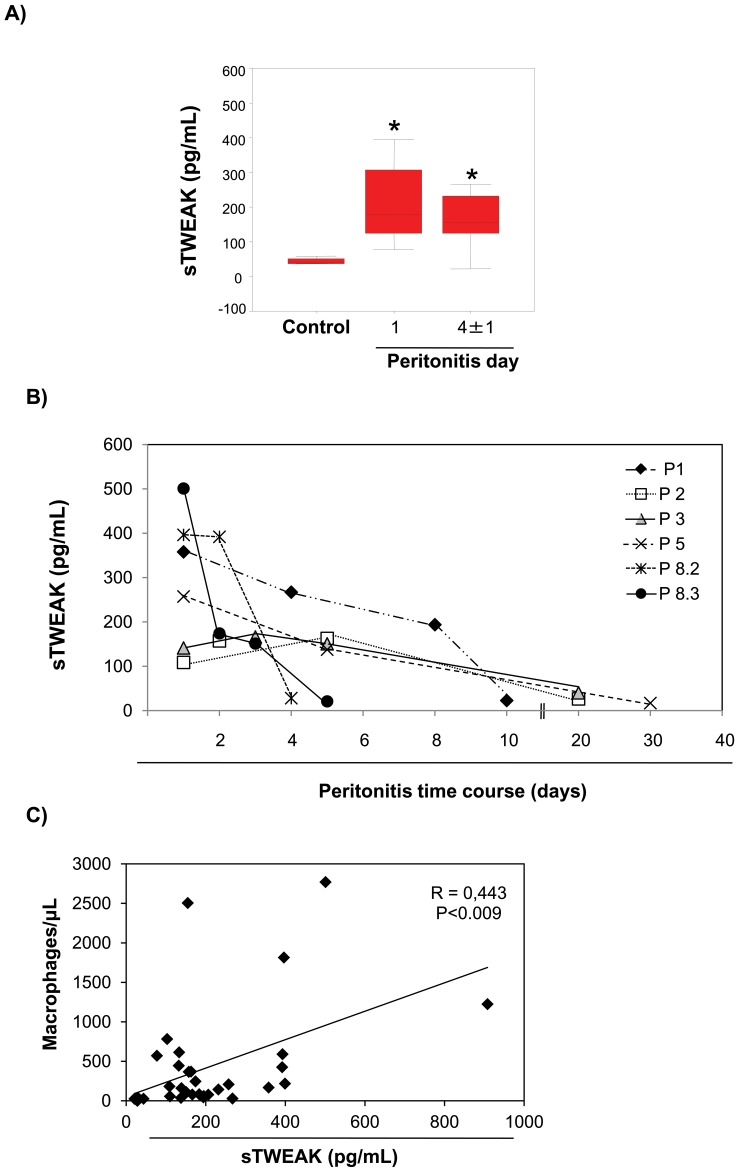
Increased peritoneal effluent sTWEAK in PD peritonitis. **A**) sTWEAK levels were measured in peritoneal effluents from PD patients with peritonitis (days 1 and 4±1) or without peritonitis. Clinical data in tables 1 and 2. *p<0.002 vs non-peritonitis. **B**) Evolution of sTWEAK levels through the peritonitis episode in 6 individual patients with at least three samples that cover the whole peritonitis episode. sTWEAK levels decrease when peritonitis resolves. Peritonitis episodes correspond to those presented in table 1. **C**) Scatter plot showing the significant positive correlation between sTWEAK levels and the number of peritoneal macrophages in peritoneal effluents during 17 episodes of peritonitis in PD patients.

sTWEAK levels were measured in peritoneal effluents from PD patients during and after peritonitis episodes. General characteristic of the studied populations are summarized in **tables 1** **and** **2**. Peritoneal effluent sTWEAK levels were increased in patients with peritonitis between day 1 and day 4±1 compared with patients without peritonitis (**Figure 1.A**). Moreover, peritoneal effluent sTWEAK levels decreased when peritonitis was resolving (**Figure 1.B**) and correlated with the number of peritoneal effluent macrophages (**Figure 1.C**). However, no correlation was found between sTWEAK and peritoneal effluent neutrophils, indeed the number of peritoneal effluent neutrophils rapidly decreases after initiation of antibiotic therapy in PD patients (**Figure S1**).These data indicate that sTWEAK could be a biomarker for severity of local inflammation in a model of human infection, peritonitis. TNFα levels were undetectable in these samples (not shown).

### Fn14 is expressed by cultured human mesothelial cells and by PD peritoneal effluent leukocytes

**Figure 2:**
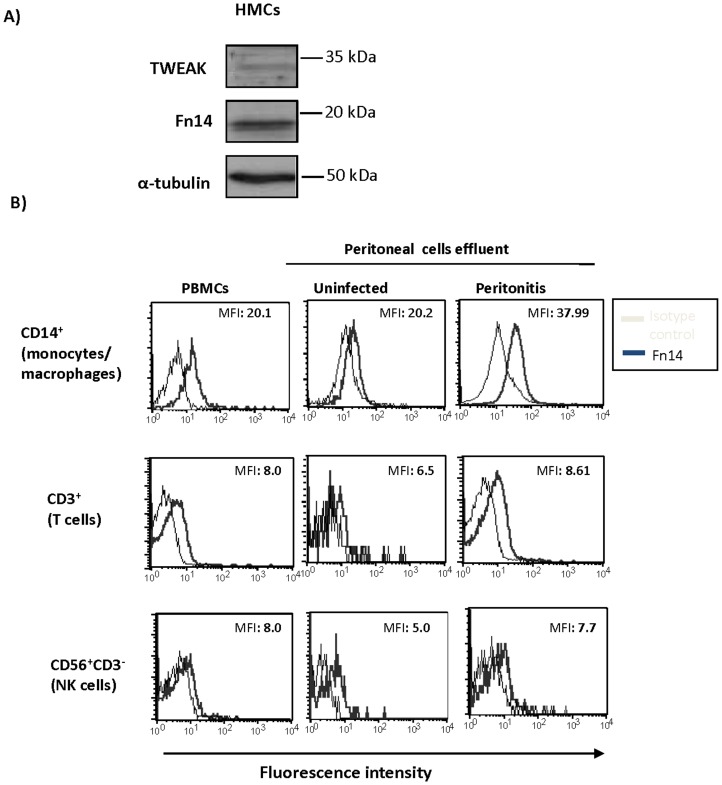
Fn14 is expressed by cultured human mesothelial cells and by leukocytes in peritoneal effluents from PD patients. **A**) Cultured human mesothelial cells (HMC) express Fn14 and TWEAK as assessed by western blot. Representative images of three different experiments. **B**) Fn14 expression was analyzed by flow cytometry in peripheral blood and peritoneal effluent leucocytes. Cells were stained with Fn14-PE and monocyte/macrophages, T cells and NK cells were identified with anti-CD14, anti-CD3 and anti-CD56 antibodies respectively within the appropriate gates according to FSC and SSC parameters. Controls for the technique were stained with isotype immunoglubulin. Peripheral blood mononuclear cells (PBMCs) were used as positive controls for leukocyte population markers. Numbers within histograms indicate mean fluorescence intensity (MFI) for Fn14 staining. Note increased Fn14 expression mainly in peritoneal macrophages from patients with peritonitis.

Fn14 is the only known signal-transducing TWEAK receptor. Thus, we evaluated cell types potentially responsive to TWEAK by assessing the expression of Fn14. Cultured human mesothelial cells express Fn14 (**Figure 2.A**). Mesothelial cells may also be a source of TWEAK (**Figure 2.A**). In addition, Fn14 expression is increased in monocytes/macrophages (CD14^+^) present in peritoneal effluents from PD patients with peritonitis compared to patients without peritonitis (**Figure 2.B**). This result suggests that mesothelial cells or monocytes/macrophages might be responsive to TWEAK during peritonitis.

### Increased Fn14 expression in peritoneal biopsies from chronic PD patients

**Figure 3:**
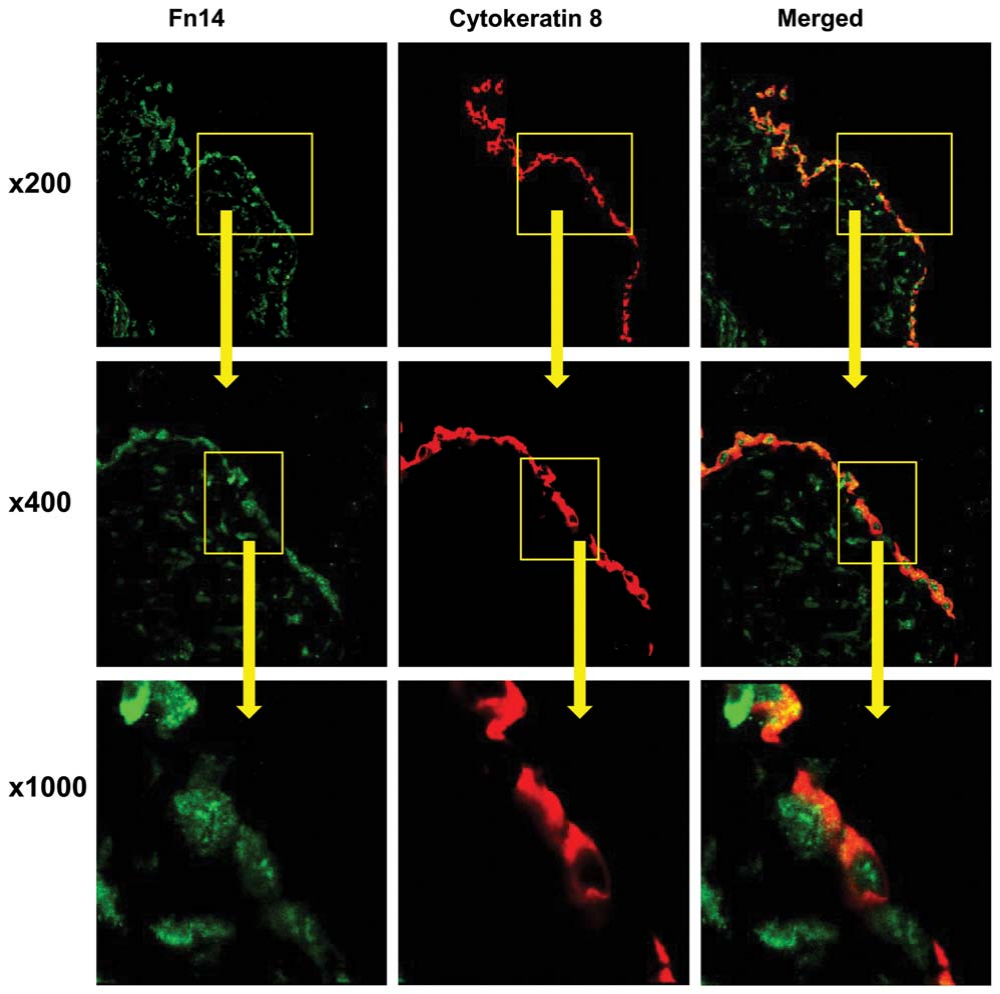
Colocalization of Fn14 and mesothelial cell markers in human preserved peritoneum. Human non-infected peritoneum from stable chronic PD patient with preserved mesothelium was stained with both anti-Fn14 antibody (green) and anti-cytokeratin 8 antibody (red). Mesothelial cells (red) express Fn14 (green). Confocal microscopy. Original magnification x200, detail x400 and x1000.

**Figure 4:**
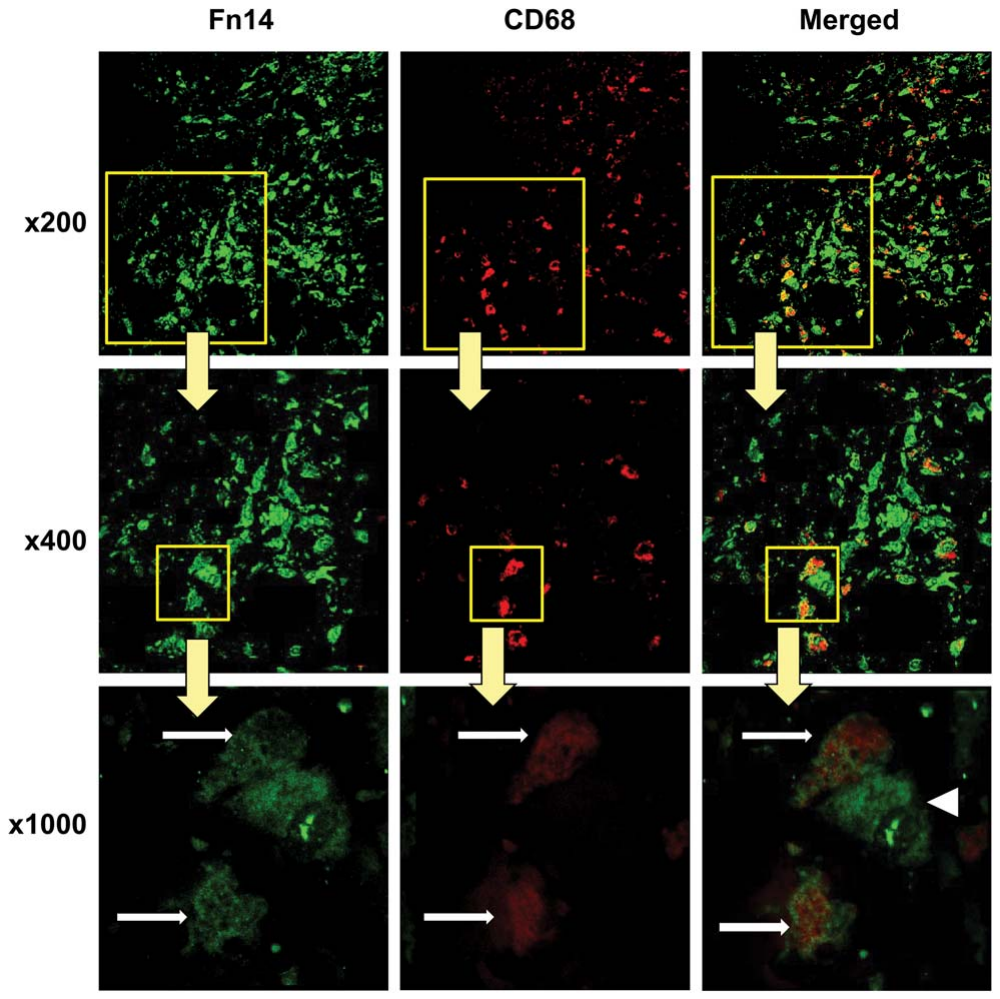
CD68 positive macrophages express Fn14 in human injured peritoneum. Human peritoneum biopsies from non-infected, stable, chronic PD patients were stained with both anti-Fn14 antibody (green) and anti-CD68 antibody (red). CD68^+^ macrophages (red) are among the submesothelial cells that express Fn14 (green)(white arrows). Additional submesothelial cells express Fn14 (arrowhead). Confocal microscopy. Original magnification x200, detail x400 and x1000.

Next we checked whether the cell culture observation of Fn14 expression by mesothelial cells was relevant in vivo. We immunolocalized Fn14 in peritoneal biopsies from subjects in table 3. Fn14 colocalized with mesothelial cell markers in peritoneal biopsies with preserved histological features, including a preserved mesothelial monolayer (**Figure 3**), confirming the cell culture observation that mesothelial cells express Fn14 (**Figure 2.A**). However, in human PD-induced peritoneal injury, Fn14 colocalized with CD68 positive macrophages and was not present in the demesothelized peritoneal membrane (**Figure 4**). Additional submesothelial cell types also expressed Fn14.

**Figure 5:**
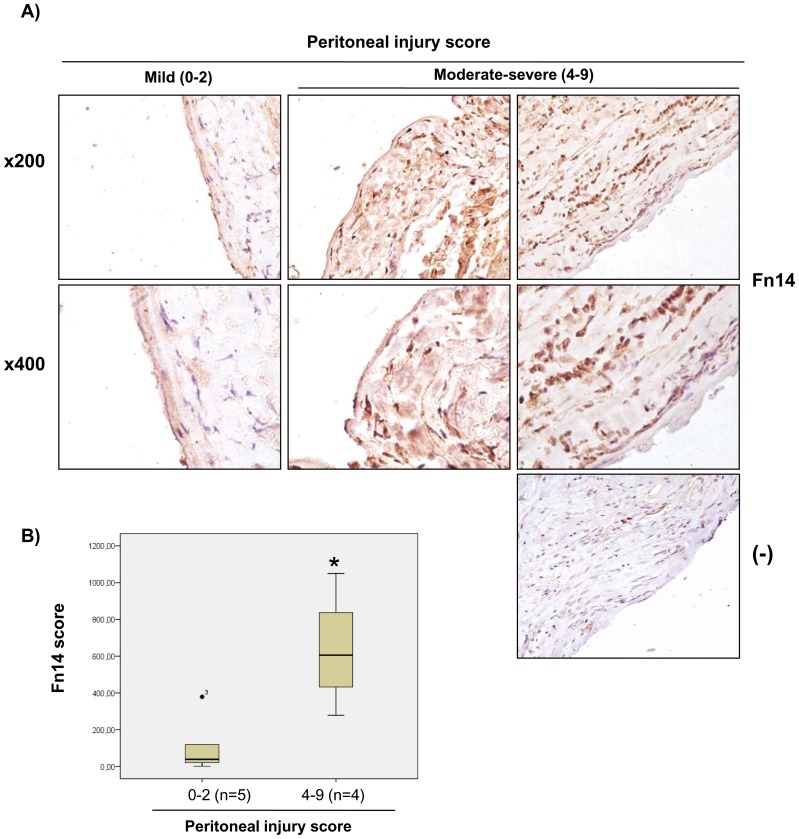
Increased Fn14 protein expression in peritoneal biopsies from non-infected patients. **A**) Fn14 immunohistochemistry in peritoneal tissue from non-infected, stable, chronic PD patients with different degrees of injury. Representative images from each group. Controls for the technique are stained with nonspecific immunoglobulin (Ig (-)). **B**) Quantification of Fn14 protein expression in peritoneal biopsies from non-infected patients. Fn14 staining was higher in patients with a more severe peritoneal histological injury score that evaluated mesothelial integrity, peritoneal fibrosis and peritoneal inflammation (**table 3**)^32^. Mean ± SEM *p<0.04, n = 4–5 patients per group.

**Figure 6:**
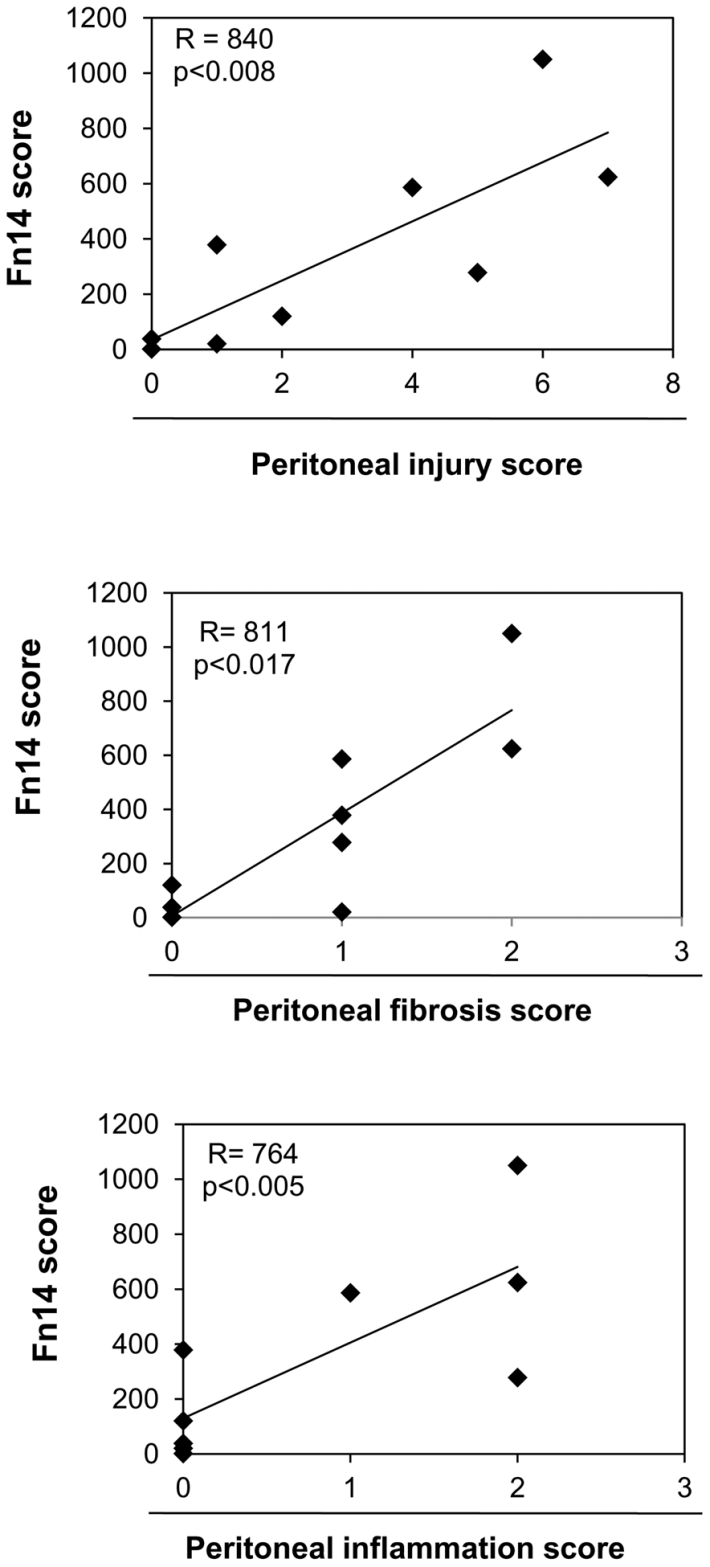
Fn14 protein expression in peritoneal tissue correlates with peritoneal injury, fibrosis and inflammation in humans. Scatter plot showing the significant positive correlation between Fn14 expression in peritoneal tissue from non-infected patients (n = 9) and histological peritoneal injury, peritoneal fibrosis and peritoneal inflammation scores.

Next, we quantified Fn14 expression in peritoneal biopsies. Peritoneal tissue samples were classified by a histological peritoneal injury score that quantified mesothelial integrity, fibrosis and inflammation (**Table 3**). Histological tissue injury was classified as mild (peritoneal injury score 0-2) or moderate-severe (score 4–9). Local peritoneal Fn14 expression was increased in patients with peritoneal injury (**Figure 5**). Fn14 expression correlated with peritoneal injury score (a composite of mesothelial integrity, peritoneal fibrosis and peritoneal inflammation), peritoneal fibrosis and peritoneal inflammation (**Figure 6**).

### Intraperitoneal TWEAK promotes peritoneal inflammation in mice

**Figure 7:**
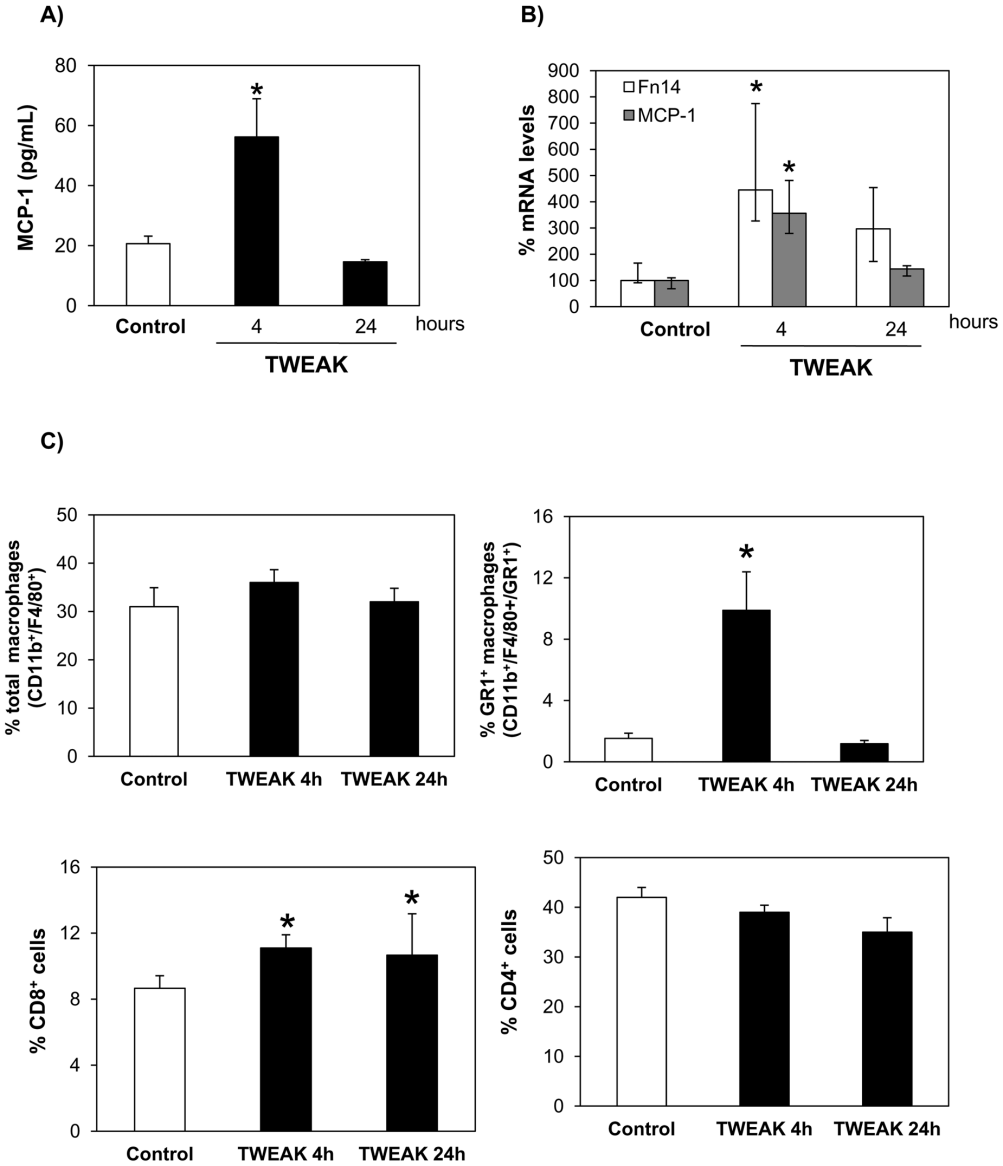
TWEAK increases inflammatory mediators in murine peritoneal effluents. **A**) MCP-1 protein levels were quantified by ELISA in peritoneal lavage of mice that had received intraperitoneal TWEAK or vehicle (control). Mean ± SEM of 5 animals per group. *p<0.001 vs control. **B**) MCP-1 and Fn14 mRNA levels measured in peritoneal lavage cells. Median (25th to 75th percentile) of 5 animals per group. *p<0.03. **C**) Quantification by flow cytometry of inflammatory cells present in murine peritoneal lavage. Mean ± SEM of 5 animals per group. *p<0.05. Macrophages were defined as CD11b+F4/80+ cells and Gr1+ macrophages as CD11b+F4/80+Gr1+ cells.

The association between TWEAK/Fn14 expression and inflammation in humans begets the question whether TWEAK promotes inflammation or inflammation increases TWEAK. We next explored TWEAK actions on the peritoneal effluent and the peritoneal tissue in vivo. Intraperitoneal TWEAK administration to mice increased peritoneal lavage monocyte chemotatic protein 1 (MCP-1) levels (**Figure 7.A**), peritoneal lavage cell expression of MCP-1 and Fn14 mRNA (**Figure 7.B**), and the percentage of Gr1^+^ macrophages, the murine equivalent to activated human CD14^+^ macrophages that express the CCR2 receptor for MCP-1 [28] (**Figure 7.C**). Moreover, TWEAK mildly increased the percentage of CD8^+^ cells (**Figure 7.C**) and neutrophils (**Figure S2**). Changes in peritoneal cell distribution or chemokine levels following a single TWEAK injection were observed at early time points (4 hours) and had returned to baseline or were decreasing at 24 h. This time-course is consistent with previously described time-courses of TWEAK or TNF-induced inflammation in other organs such as the kidney [19].

**Figure 8:**
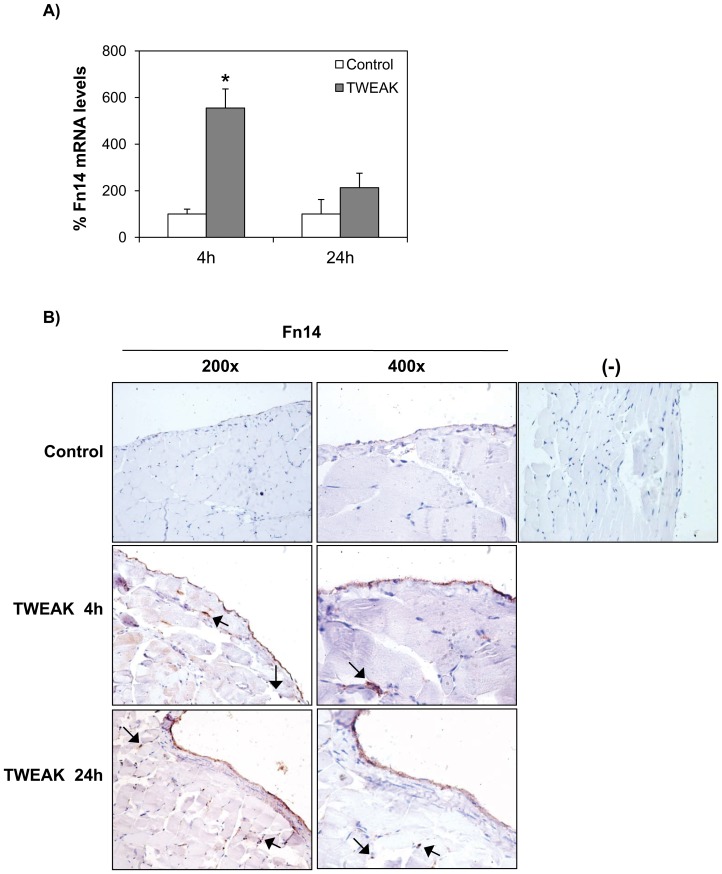
TWEAK increases Fn14 expression in murine peritoneal tissue in vivo. **A**) Intraperitoneal TWEAK administration increased Fn14 mRNA in peritoneal tissue. Mean ± SEM of 5 animals per group. *p<0.02. **B**) Fn14 immunohistochemistry. TWEAK administration increased Fn14 expression in mesothelial cells and submesothelial infiltrating cells (arrows). Controls for the technique are stained with nonspecific Ig (-). Images representative of 5 animals per group.

**Figure 9:**
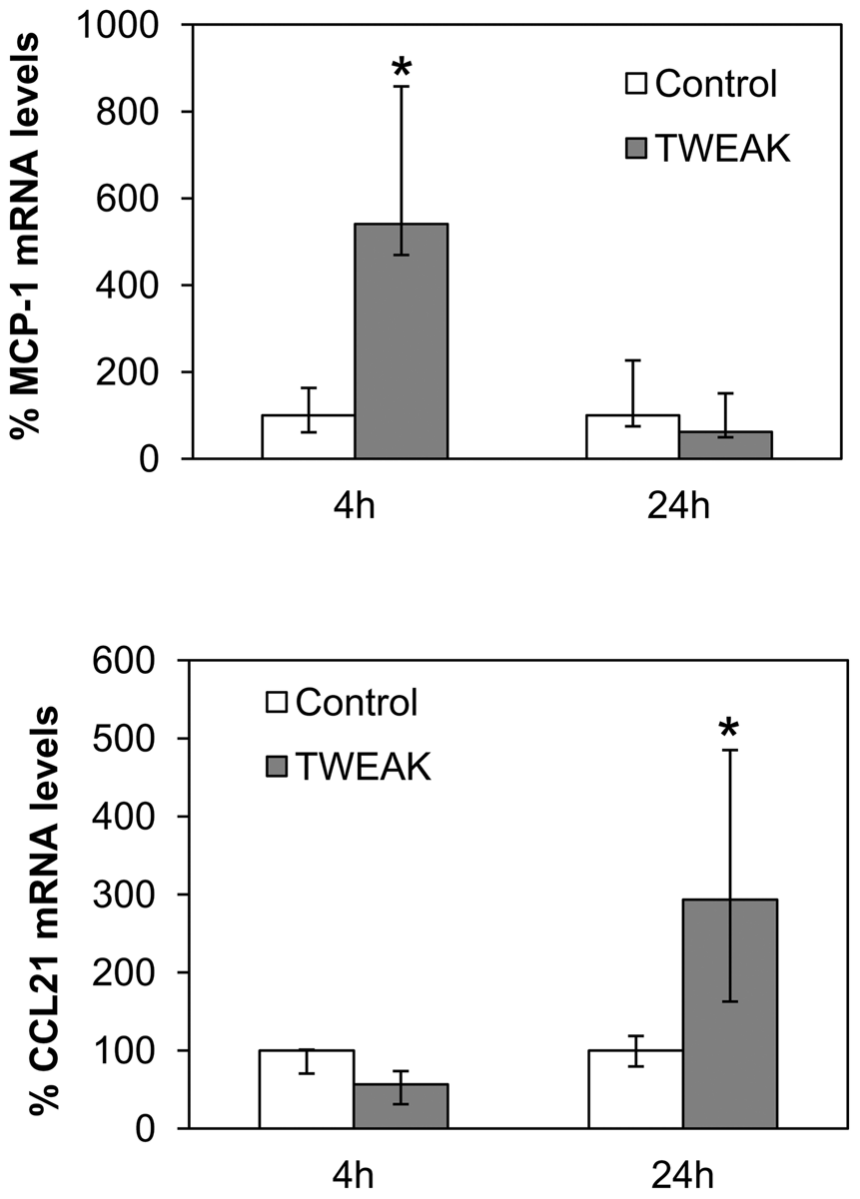
TWEAK promotes murine peritoneal tissue chemokine mRNA expression *in vivo*. MCP-1 and CCL21 mRNA levels measured by RT-PCR in murine peritoneal tissue 4 and 24 hours following TWEAK administration. Median (25th to75th percentile) of 5 animals per group. *p<0.05.

**Figure 10:**
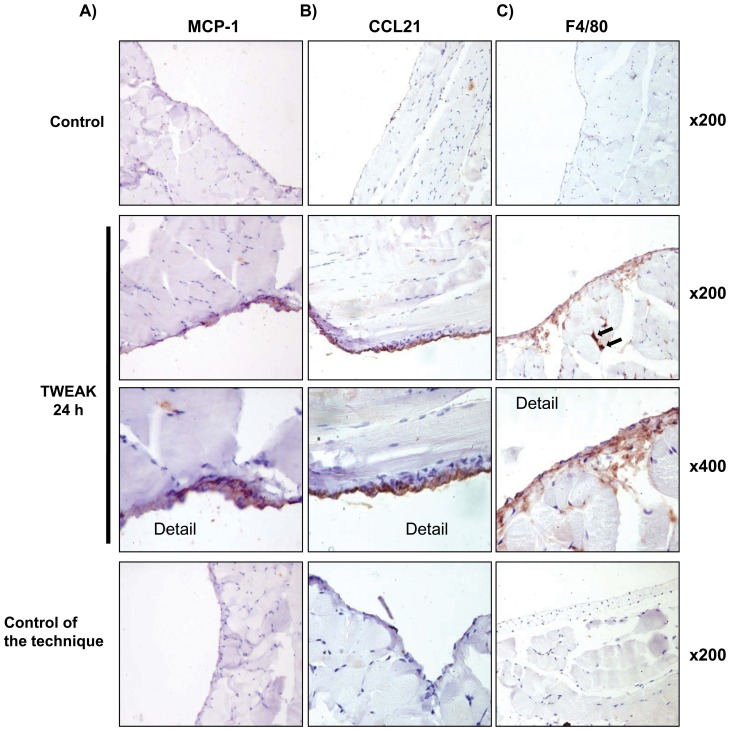
TWEAK induces chemokine protein expression and peritoneal inflammation in mice *in vivo*. **A and B**) Increased MCP-1 (**A**) and CCL21 (**B**) protein expression in mesothelium from mice 24 h after TWEAK injection. **C**) F4/80 antigen immunohistochemistry. An increased number of submesothelial macrophages stained with F4/80 were noted 24 h after TWEAK injection (arrows). Controls for the technique are stained with nonspecific Ig. Images representative images of 5 animals per group.

TWEAK also increased peritoneal tissue Fn14 mRNA expression as assessed by quantitative RT-PCR (**Figure 8.A**). Immunohistochemistry showed Fn14 expression in mesothelial cells and in infiltrating submesothelial cells (**Figure 8.B**), as was the case for human peritoneal biopsies (**Figure 3** **,** **4**). TWEAK also increased peritoneal tissue MCP-1 and CCL21 mRNA expression (**Figure 9**). These chemokines were chosen for analysis because they represent canonical and non-canonical NFKB activation by TWEAK, respectively [29], and they are relevant for peritoneal injury since MCP-1 attracts macrophages and CCL21 attracts fibrocytes [30]. Upregulation of peritoneal tissue Fn14 and MCP-1 mRNA was observed earlier (4 hours) than CCL21 mRNA upregulation (24 hours). This time course is similar to that observed in the kidney of TWEAK-treated mice [19], [20]. Likewise, immunohistochemistry disclosed increased MCP-1 and CCL21 protein in peritoneal tissue of mice treated with TWEAK (**Figure 10**). Mesothelial cells expressed the chemokines, indicating that these Fn14-expressing cells are key peritoneal responders to TWEAK stimulation. Moreover, TWEAK promoted tissue macrophage recruitment (**Figure 10**). Macrophage infiltration was observed in the sub-mesothelial region, next to Fn14- and chemokine-expressing mesothelial cells.

## Discussion

The main findings are high sTWEAK levels in peritoneal effluents during human peritonitis, a correlation of peritoneal tissue Fn14 receptor expression with peritoneal injury in long-term PD, and the observation that in vivo TWEAK activates Fn14-expressing cells mesothelial cells and promotes peritoneal inflammation in mice. These findings identify sTWEAK as a potential biomarker and therapeutic target in peritoneal inflammation. This observation may be of interest to other forms of bacterial peritonitis and to chronic peritoneal injury associated with PD. Moreover, the findings could apply to tissue injury in the course of other human bacterial infections.

The nature of PD allows repeated non-invasive access to events taking place in the peritoneal cavity. Thus, PD is a human model which allows the monitoring of inflammatory events in vivo during infection or in the long-term course of sterile inflammation. Findings in the human PD model may be representative of events in other conditions characterized by bacterial infection or sterile inflammation and may guide the development of hypothesis and studies on the role of specific molecules in these events. We have taken advantage of this model to identify high levels of sTWEAK in peritoneal effluents during peritonitis episodes and to study the potential role of en excess of this cytokine in the peritoneal cavity.

While our study draws attention to the potential of sTWEAK as a biomarker in peritoneal inflammation, the design does not allow definition of a specific role of sTWEAK in monitoring of peritoneal inflammation and in assessing potential outcomes that might guide therapeutic decision-making regarding intensification or change of antibiotic therapy or the decision to remove the peritoneal catheter. However, further studies should address these possibilities. A role in this respect may be envisioned since another candidate indicator of the severity of inflammation, TNFα, could not be detected in these samples. We did not address the sources of peritoneal effluent sTWEAK in humans in vivo, but cell culture results presented here and a literature review suggest that both mesothelial cells and macrophages are potential sources [31].

Inflammation is a physiological response to bacterial aggression. In the course of peritonitis multiple inflammatory mediators are released locally [32]–[35]. For some of them functional evidence of their link to tissue injury has been provided [23], [36]–[40]. Thus, TGFβ1 promotes peritoneal fibrosis and dysfunction [37], [39], PDGF-B promotes angiogenesis [38], IL-6 and its soluble receptor (sIL-6R) control the pattern of leukocyte recruitment during peritoneal inflammation [40] and the IFNγ/TNFα combination promotes mesothelial cell apoptosis [23]. Indeed, a single episode of very severe peritonitis may cause irreversible peritoneal fibrosis. It is thus necessary to understand the different contributors to peritoneal inflammation. Our results indicate that during peritoneal inflammation mesothelial cells upregulate the TWEAK receptor Fn14 and that TWEAK activates mesothelial cells to promote the expression of chemokines dependent on both canonical and non-canonical NFκB activation [19], [20] to attract macrophages into the peritoneal cavity and peritoneal tissue. Although we did not specifically explore non-canonical vs canonical NFκB activation in the present work, the time-course of gene expression of the canonical NFκB target MCP-1 and of the non-canonical NFκB target CCL21 observed in vivo in mice is consistent with prior studies supporting the involvement of these pathways in upregulation of these specific chemokines [20]. Indeed, the transient, self-limited nature of MCP-1 upregulation is characteristic of canonical NFκB response elicited by a single exposure to TWEAK or TNF [29]. Macrophages are the key targets of the chemotactic activity of MCP-1. In this regard, the observed increase in the percentage of Gr1^+^ peritoneal lavage macrophages coinciding with peak MCP-1 levels is consistent with the inflammatory nature of these macrophages. Gr1^+^ macrophages express the MCP-1 receptor CCR2 and activate integrins, increasing their ability to roll on endothelial cell and migrate into inflamed tissue [41]. TWEAK may also directly activate macrophages, since Fn14 expression is upregulated in macrophages during peritonitis. Among CCL21 targets, fibrocytes are of particular interest. These are circulating fibroblast precursors that are attracted by CCL21 and may contribute to peritoneal fibrosis [30]. In this regard, Fn14 expression in human peritoneum correlated with the peritoneal fibrosis score. In mice, intraperitoneal TWEAK also transiently increased peritoneal lavag neutrophils, although the effect was milder than on macrophages. In this regard, no correlation between peritoneal effluent sTWEAK and macrophages was observed in human PD peritonitis, suggesting that factors other than TWEAK are more important in recruiting neutrophils or that neutrophil contribution to sTWEAK levels is minor.

In several animal models TWEAK has consistently shown to promote further inflammation and tissue injury during sterile inflammation. This has been observed in kidney, vascular and central nervous system disease [15]–[20], [42], [43]. The association between Fn14 expression and histological peritoneal injury scores in chronic, non-infected PD patients suggests that TWEAK may also have such a role in long-term PD peritoneal injury. Current ongoing clinical trials are testing the tissue protective action of neutralizing anti-TWEAK antibodies during sterile inflammation (http://clinicaltrials.gov/show/NCT00771329, accessed on March 23, 2013, http://clinicaltrials.gov/ct2/show/NCT01499355, accessed on October 23, 2013). The results of these trials will guide the potential evaluation of this therapeutic tool in the PD context. In this regard, TWEAK has additional actions of potential relevance for peritoneal injury which we have not addressed. Thus, TWEAK has a pro-angiogenic role and angiogenesis has been associated with peritoneal dysfunction. However, the role of TWEAK/Fn14 during infection has not been adequately characterized. A potential contribution of sTWEAK to peritoneal defense during acute peritonitis, probably by favoring leukocyte recruitment, cannot be excluded.

In conclusion, TWEAK and its receptor are highly expressed in the peritoneum under diverse pathological circumstances and they promote inflammation. Further studies are needed to explore the biomarker and therapeutic potential of these findings.

## Supporting Information

Figure S1 **Human peritoneal effluent sTWEAK levels do not correlate with peritoneal effluent neutrophils. A)** Neutrophil counts rapidly decrease in peritoneal effluents from PD patients after initiation of antibiotic therapy. Mean ± SEM. Clinical data in table 1. *p<0.002 vs day 1. **B)** Scatter plot showing the negative correlation between sTWEAK levels and the number of peritoneal neutrophils in peritoneal effluents during 17 episodes of peritonitis in PD patients.
(TIF)

Figure S2 **TWEAK induces transient neutrophil recruitment in murine peritoneal effluents**. Quantification by flow cytometry of neutrophils present in murine peritoneal lavage. Mean ± SEM of 5 animals per group. *p<0.008 vs control. Neutrophils were defined as Gr1^+^/F4/80^−^cells.
(TIF)
