# Supplementary figures and images for: TWEAK Promotes Peritoneal Inflammation
Source: PLoS One. 2014 Mar 5;9(3):e90399. doi: 10.1371/journal.pone.0090399 (PMC3944020; doi:10.1371/journal.pone.0090399)

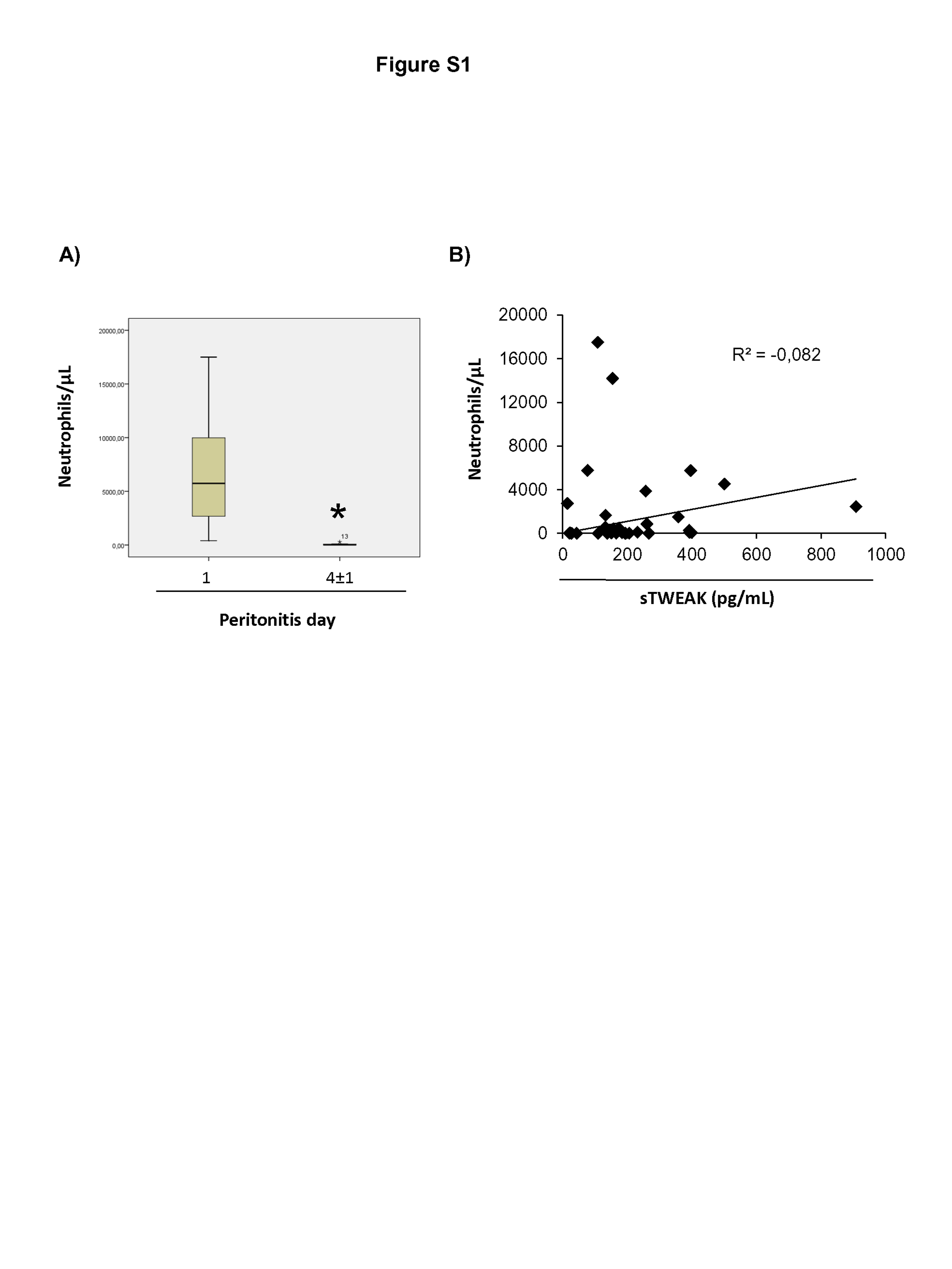

Supplement: Figure S1 — Human peritoneal effluent sTWEAK levels do not correlate with peritoneal effluent neutrophils. A) Neutrophil counts rapidly decrease in peritoneal effluents from PD patients after initiation of antibiotic therapy. Mean ± SEM. Clinical data in table 1. *p<0.002 vs day 1. B) Scatter plot showing the negative correlation between sTWEAK levels and the number of peritoneal neutrophils in peritoneal effluents during 17 episodes of peritonitis in PD patients. (TIF) [file pone.0090399.s001.tif]

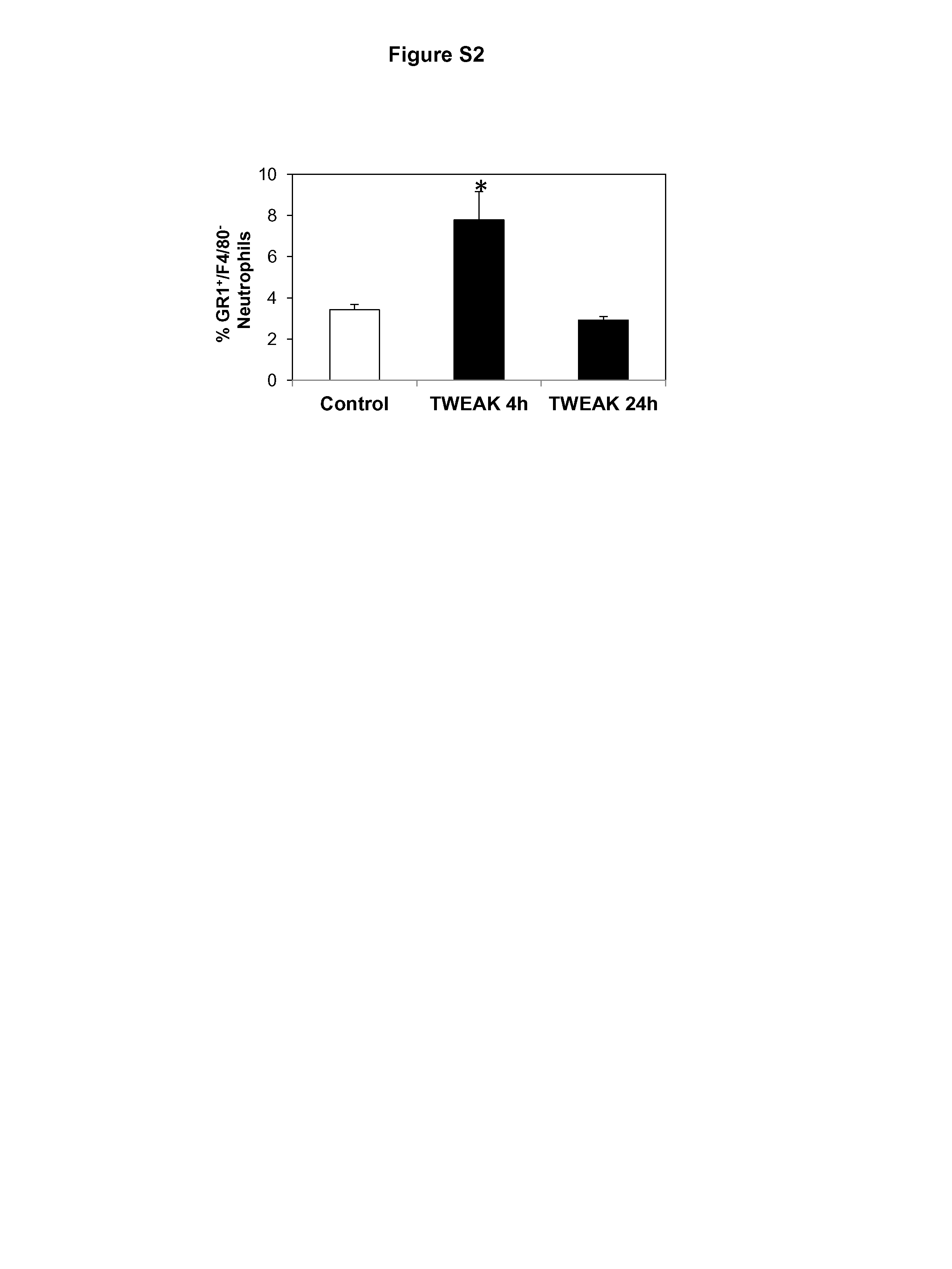

Supplement: Figure S2 — TWEAK induces transient neutrophil recruitment in murine peritoneal effluents. Quantification by flow cytometry of neutrophils present in murine peritoneal lavage. Mean ± SEM of 5 animals per group. *p<0.008 vs control. Neutrophils were defined as Gr1+/F4/80−cells. (TIF) [file pone.0090399.s002.tif]
